# Experience-dependent plasticity in white matter microstructure: reasoning training alters structural connectivity

**DOI:** 10.3389/fnana.2012.00032

**Published:** 2012-08-22

**Authors:** Allyson P. Mackey, Kirstie J. Whitaker, Silvia A. Bunge

**Affiliations:** ^1^Helen Wills Neuroscience Institute, University of California at BerkeleyBerkeley, CA, USA; ^2^Department of Psychology, University of California at BerkeleyBerkeley, CA, USA

**Keywords:** cognitive training, fluid reasoning, plasticity, diffusion-weighted imaging, test preparation

## Abstract

Diffusion tensor imaging (DTI) techniques have made it possible to investigate white matter plasticity in humans. Changes in DTI measures, principally increases in fractional anisotropy (FA), have been observed following training programs as diverse as juggling, meditation, and working memory. Here, we sought to test whether three months of reasoning training could alter white matter microstructure. We recruited participants (*n* = 23) who were enrolled in a course to prepare for the Law School Admission Test (LSAT), a test that places strong demands on reasoning skills, as well as age- and IQ-matched controls planning to take the LSAT in the future (*n* = 22). DTI data were collected at two scan sessions scheduled three months apart. In trained participants but not controls, we observed decreases in radial diffusivity (RD) in white matter connecting frontal cortices, and in mean diffusivity (MD) within frontal and parietal lobe white matter. Further, participants exhibiting larger gains on the LSAT exhibited greater decreases in MD in the right internal capsule. In summary, reasoning training altered multiple measures of white matter structure in young adults. While the cellular underpinnings are unknown, these results provide evidence of experience-dependent white matter changes that may not be limited to myelination.

## Introduction

Advances in neuroimaging techniques have led to important progress in understanding how brain regions are structurally and functionally connected in the human brain. Much of this knowledge has been obtained from cross-sectional studies, which provide only a snapshot of an individual's brain at a single point in time. As a result, we have only just begun to understand how learning and experience shape brain connectivity. In this paper, we provide evidence for experience-dependent changes in white matter microstructure among young adults participating in intensive cognitive training.

White matter microstructure can be investigated *in vivo* using diffusion-weighted imaging (DWI). DWI relies on the biophysical principal that, as water diffuses, it follows the path of least resistance. Water diffusing in any given white matter voxel encounters axons (which contain dense cytoskeletons, are bounded by cellular membranes, and are surrounded by myelin) and glial cells. Research in animals has shown that water preferentially moves along axons rather than through the myelin sheath (for review see Beaulieu, 2002; Assaf and Pasternak, 2008). Activity-dependent increases in myelination could, therefore, reduce diffusion through the myelin sheath. However, changes in unmyelinated axons, and the number and/or size of glia, could also alter diffusion.

Diffusion tensor imaging (DTI) analysis fits a tensor to DWI to extract measures of axial diffusion [axial diffusion (AD or λ_1_)], the preferential direction of water diffusion, and radial diffusion (RD or λ_23_), the average of the two directions perpendicular to AD. AD has been related to diffusion along an axon, whereas RD is linked to diffusion through the myelin sheath (Beaulieu, 2002). Fractional anisotropy (FA) is a scaled ratio of AD to RD (Basser, 1995; Pierpaoli and Basser, 1996). High FA indicates strong directionality of water diffusion, i.e., high white matter coherence. Mean diffusivity (MD) is the average of diffusion parameters in all three orthogonal directions. Low MD reflects a high density of cells and/or extracellular material that impedes the diffusion of water through brain tissue. Because these diffusion measures (AD, RD, FA, and MD) have been shown to relate to different aspects of white matter composition (Song et al., 2002, 2003), some DTI studies of neuroplasticity have investigated the measures separately, though many have focused specifically on FA.

Neuroplasticity in humans has been studied through two main approaches. A first approach has been to compare experts to novices, with the assumption that any brain differences between the groups can be attributed to the extensive training experts have received over the course of their lives. This work has yielded mixed results in terms of the direction of observed differences in DTI measures. For example, when comparing musicians to non-musicians, both increased and decreased FA in the corticospinal tract have been observed (Imfeld et al., 2009). Additionally, when comparing fighter pilots—who demonstrate enhanced cognitive control relative to the general population—with controls, lower RD in white matter underlying parietal cortex and higher RD in white matter near medial frontal cortex were observed (Roberts et al., 2010). In such studies, it is not possible to disambiguate the effects of experience from an innate predisposition to pursue a particular type of training.

A second approach to studying neuroplasticity in humans involves direct experimental control over individuals' experience. To date, there have been very few published studies on training-related plasticity in white matter microstructure in healthy adults. One study showed that working memory training increased FA in left parietal and frontal white matter, as well as white matter under somatomotor cortices (Takeuchi et al., 2010). However, this study did not include a control group, so effects of maturation in the study's young participants cannot be ruled out. A second study showed that juggling training increased FA in white matter near right posterior parietal cortex, potentially related to enhanced use of visual areas important for detecting motion (Scholz et al., 2009). A third study showed decreased FA in bilateral frontal lobes, and increased MD in right parietal lobe and cerebellum following practice with a balancing task (Taubert et al., 2010). Finally, a fourth study showed that meditation training leads to increased FA in medial anterior corona radiata (Tang et al., 2010). Further analysis of this data set revealed that the majority of voxels exhibiting increased FA showed both decreased RD and AD (Tang et al., 2012).

In the present study, we investigated white matter changes associated with intensive training on relational reasoning, the ability to compare and combine mental representations. The reasoning training paradigm consisted of a course aimed at improving scores on the Law School Admission Test (LSAT). The LSAT has three parts: Logic Games, Logical Reasoning, and Reading Comprehension (for a sample test, see http://www.lsac.org/jd/pdfs/SamplePTJune.pdf). Both of the logic sections heavily tax relational reasoning. Because this exam plays an almost determinative role in law school acceptance, we reasoned that students would be highly motivated to prepare for it.

Numerous studies have implicated a bilateral fronto-parietal network in reasoning (see Hampshire et al., 2011; Prado et al., 2011; Krawczyk, 2012 for review), several of which have suggested that rostrolateral prefrontal cortex (RLPFC) is specifically involved in relational integration (Wendelken and Bunge, 2010; Hampshire et al., 2011; Wendelken et al., 2011a,b). Based on these findings, we predicted changes in white matter connecting frontal and parietal cortices both within and between hemispheres. We were specifically interested in changes in the trained group that were significantly greater than those measured for an age- and IQ-matched control group. In other words, we considered changes in the trained group that could not be accounted for by typical development in young adults over 3 months to be the strongest evidence for experience-dependent plasticity.

## Methods

### Participants

Twenty-five adults (14 females) took part in the training group, and twenty-five adults (14 females) took part in the age- and IQ-matched pre-law control group. The training group was recruited through e-mail and classroom announcements to students in Blueprint Test Preparation courses. The control group was recruited through e-mails to pre-law organizations and online postings. Recruitment and experimental procedures were approved by the Committee for the Protection of Human Subjects at the University of California at Berkeley. Participants had no history of psychiatric or neurological disorder, and were fluent in English. Three participants in the trained group and two participants in the control group were left-handed.

Two participants—one from each group—were excluded from the study because they exhibited dramatic changes in stress levels and amount of sleep from time 1 to time 2 (more than three SD from the mean of all participants). Additionally, two participants from the control group were excluded because more than 5% of their brain volumes contained movement-related artifacts. Finally, we tested for outliers in average whole-brain diffusion measures at time 1, time 2, and in change between time points, and excluded one participant in the trained group for showing a decrease in MD and RD that was greater than two standard deviations lower than the mean across both groups. Thus, our final dataset included DTI data at two time points for 23 trained individuals and 22 controls (Table 1).

**Table 1:** **Demographic and behavioral measures for study participants**.

	**Trained *N* = 23**	**Control *N* = 22**
Age	21.39 (1.42)	21.44 (2.15)
WASI Matrix	29.75 (2.10)	29.37 (1.74)
WASI Vocabulary	66.33 (5.76)	67.10 (3.67)
Days between scans	89.17 (15.61)	90.91 (22.87)
**PERCEIVED STRESS**		
Time 1	21.67 (7.71)	20.24 (7.32)
Time 2	21.16 (7.07)	22.11 (9.13)
**HOURS OF SLEEP**		
Time 1	7.50 (0.88)	7.57 (0.96)
Time 2	7.33 (1.08)	7.34 (1.14)

Means and standard deviations are reported. None of the measures differed significantly between groups (P > 0.2).

### Behavioral data

During the first testing session, we administered the Young Adult Self Report (Achenbach, 1990, 1997) to screen out participants who scored in the clinical range. We also administered two scales from the Wechsler Adult Scale of Intelligence (WASI) (Wechsler, 1999), Matrix Reasoning and Vocabulary, to match the groups on IQ (see Table 1). During both testing sessions, we administered the Perceived Stress Scale (Cohen et al., 1983) and asked participants to report their sleep schedules for the preceding two weeks. Reported stress levels and hours of sleep did not differ between groups at either time point (*Ps* > 0.4), and neither group changed significantly between time points (*Ps* > 0.2).

### Training paradigm

We selected the Blueprint Test Preparation course as the training paradigm because it provided more classroom time than other local programs: 100 h distributed across the three components of the LSAT (35 h for Logic Games, 35 h for Logical Reasoning, and 30 h for Reading Comprehension). “Logic Game” questions require test takers to integrate a series of rules in order to sequence or group a set of items. “Logical Reasoning” questions ask them to determine the logical flaw in an argument, identify an assumption, or choose a statement that would strengthen or weaken an argument. The remaining 30 h of class time were dedicated to “Reading Comprehension” questions that require test-takers to interpret short passages of text.

For the Logic Games section, students were taught to break down problems into the essential information and to use diagrams to represent and integrate rules. For the Logical Reasoning section, students were taught basic logic principles (such as *modus ponens* and *modus tollens*), as well as how to avoid common logical fallacies. Students attempted problems at home and then instructors worked through the problems in class, answering any questions students might have. Special attention was paid to keeping motivation levels high by making the content fun through relatable examples.

Four LSAT practice tests were administered throughout the course. Practice test scores were provided either by the participants or (with participants' consent) by the test preparation company. We compared the scores on each of the LSAT sections for the first and fourth practice test as an index of change from time 1 to time 2.

### Voxel-based morphometry analysis

To rule out the possibility that gray matter changes associated with training could be misinterpreted as changes in DTI parameters, we performed voxel-based morphometry analyses on the structural data from the trained group using Functional MRI of the Brain Software Library (FSL) (Ashburner and Friston, 2000; Good et al., 2001; Smith et al., 2004). Structural images were skull-stripped using Brain Extraction Tool (BET) (Smith, 2002), and tissue-type segmentation was carried out using FMRIB's Automated Segmentation Tool (FAST)4 (Zhang et al., 2001). Gray-matter partial volume images were then aligned to standard space using FSL's Linear Image Registration Tool (FLIRT) (Jenkinson and Smith, 2001; Jenkinson et al., 2002), followed by nonlinear registration using FMRIB'S Nonlinear Image Registration Tool (FNIRT) (Andersson et al., 2007a,b), which uses a b-spline representation of the registration warp field (Rueckert et al., 1999). The resulting images were averaged to create a study-specific template, to which the native grey matter images were then non-linearly re-registered. The registered partial volume images were then modulated (to correct for local expansion or contraction) by dividing by the Jacobian of the warp field. The modulated segmented images were then smoothed with an isotropic Gaussian kernel with a sigma of 4 mm. Finally, a voxel-wise paired *t*-test GLM comparing pre-training to post-training data was applied using Randomise (Nichols and Holmes, 2002) with 5000 permutations, correcting for multiple comparisons at *P* < 0.05.

### DTI data acquisition and preprocessing

Data were acquired on a three Tesla Siemens Trio TIM MR scanner using a 12-channel head coil with a maximum gradient strength of 40 mT/m. Structural and functional scans were collected in a fixed sequence across subjects and across time points. DTI data were acquired using echo-planar imaging (EPI; TR = 7900 ms; TE = 102 ms; 2.2 mm^3^ isotropic voxels; 55 axial slices). Parallel acquisition (GRAPPA) was used with at an acceleration factor of 2. Seven non-diffusion-weighted directions and 64 diffusion-weighted directions were acquired with a b-value of 2000 s/mm^2^, uniformly distributed across 64 gradient directions.

Analyses were performed using tools from FDT (Functional MRI of the Brain (FMRIB) Diffusion Toolbox, part of FSL 4.1; Smith et al., 2002; Woolrich et al., 2009). Brain volumes were skull-stripped using the BET (Smith, 2002). A 12-parameter affine registration to the *b* = 0 weighted volume was applied to correct for head motion and eddy current distortions introduced by the gradient coils, and the gradient directions were rotated accordingly. A diffusion tensor model was fitted to the data in a voxel-wise fashion to generate whole-brain maps of AD, RD, MD, and FA.

The first volume of our DTI acquisition had no diffusion weighting and was used to align the DTI scans at both time points to each other using a 12 parameter affine transformation and skull images to constrain the registration scaling using FLIRT (Jenkinson and Smith, 2001; Jenkinson et al., 2002). Both images were resampled into a space halfway between the two. This transformation was then applied to the FA maps and the aligned maps averaged to generate a subject-specific mid-space template. We subsequently non-linearly aligned these template FA maps into standard space using FNIRT (Andersson et al., 2007a,b). Whole-brain MD, AD, and RD maps were aligned to standard space through application of the same two-step transform (linearly into subject-template space, then non-linearly into standard space).

A white matter mask was created from each subject's high resolution T1-weighted scan, after brain extraction, using FAST (Zhang et al., 2001). This mask was transformed into the subject's DTI space by applying the inverse of the affine registration of the non-diffusion weighted volume to the high resolution image. Both the registration and calculations of the inverse transform used FLIRT (Jenkinson et al., 2002). Once in DTI space, the white matter masks were registered to subject-template space and combined (through multiplication) to create a subject-specific definition of white matter voxels.

### DTI analyses

We performed voxel-wise statistical analysis using TBSS (Tract-Based Spatial Statistics, Smith et al., 2006). After FA maps were aligned to standard space, the mean FA image was generated and thinned to produce a mean FA skeleton that represented the centers of all tracts common to the group. Each subject's aligned FA, AD, RD, and MD data were then projected onto this skeleton by finding the nearest maximum FA value for the individual. This projection step aims to remove the effect of cross-subject spatial variability that remains after the non-linear registration. Skeletonized difference images (time 2–time 1) were created for each subject, and the resulting data were fed into an unpaired *t*-test to compare the trained group to the control group. Voxel-wise cross-subject permutation-based nonparametric statistics were performed using Randomise (Nichols and Holmes, 2002) with 5000 permutations and threshold-free cluster enhancement to correct for multiple comparisons at *P* < 0.05 (Smith and Nichols, 2009). We used the same statistical approach to test for pre-training differences between groups (unpaired *t*-test of time 1 data).

To better characterize the anatomy of white matter showing an effect of training, we examined a recently developed tensor index used to identify regions of crossing fibers (Douaud et al., 2011): the mode of anisotropy (Ennis and Kindlmann, 2006). Regions with a positive mode have linear anisotropy, and are likely to be part of a highly directional tract. In contrast, regions with a low or negative mode can be described as having planar anisotropy, and are more likely to contain crossing fibers. We extracted mode of anisotropy values from voxels that were significant in the whole-brain analyses, as well as mode values across the entire white matter skeleton. Specifically, we extracted values from the average of all time 1 and time 2 mode maps after they had been registered into the standard space by following the same two-step registration process as described above. Histograms with a bin width of 0.01 were created using fslstats, an FSL tool (Smith et al., 2004). We used a Mann-Whitney *U*-test to investigate differences in the distributions of mode values within each of the results regions and the white matter skeleton.

We tested for correlations between LSAT improvement, as measured by the difference between the first and fourth practice test, and diffusion changes at the whole brain level following the approach described above. We then tested for brain-behavior correlations in the anatomical regions defined by the Johns Hopkins University White Matter Label Atlas (Mori, 2005). While we predicted correlations in frontoparietal white matter, we decided to perform an exploratory analysis because we considered that brain-behavior relationships might be most prominent in tracts less centrally involved in reasoning. Therefore, we tested all 48 labels and corrected all statistics for multiple comparisons using a randomization-based family-wise error correction (Nichols and Hayasaka, 2003).

White matter labels were nonlinearly registered to subject-template space (halfway between time 1 and time 2 for each subject, described above) using the inverse of the transform previously used to register subject data to standard space. Then, the average value of all voxels which lay within each ROI and the subject-specific white matter mask was extracted separately from each map. A difference measure was calculated by subtracting the average value for time 1 from the average value for time 2.

**Figure 1:**
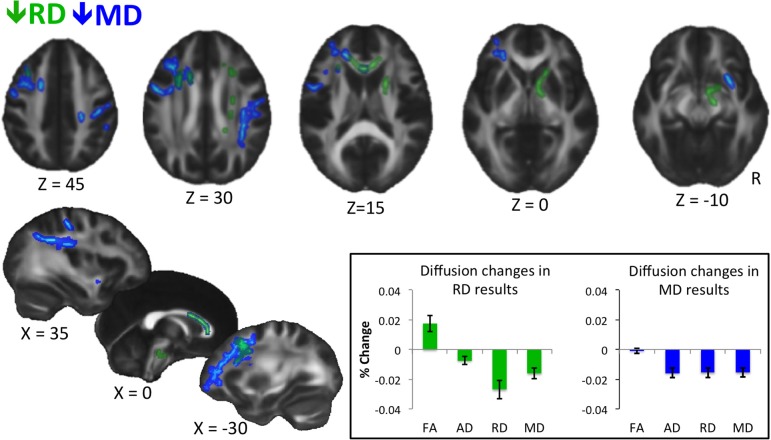
**Results of whole-brain voxel-wise statistics.** Decreases in RD are shown in green, and decreases in MD are shown in blue. Statistics were performed on skeletonized images, and results were filled for visualization purposes. Results are thresholded at *P* < 0.05, corrected for multiple comparisons with threshold-free cluster enhancement (Smith and Nichols, 2009). Inset shows percent change in diffusion measures extracted from voxels showing a significant decrease in RD (left) and MD (right) for the trained group only. Error bars represent standard error of the mean. No statistics are performed as they would be biased because values are extracted from voxels showing a significant change in diffusion at the whole brain level. The graph is meant to show qualitative differences in diffusion parameters between RD and MD results.

We also applied this process to calculate the average difference values for FA, AD, RD and MD in the voxels that reached significance in the whole brain analyses (see inset in Figure 1).

## Results

### Behavioral improvement

For participants for whom all four practice test scores were available (*n* = 16), training was associated with a gain of nine points on the LSAT (*P* < 0.001, *df* = 15, *t* = 6.59). Subtest data was available for 13 participants. These participants improved significantly on the two reasoning components of the test, Logic Games (*P* < 0.01, *df* = 12, *t* = 3.21) and Logical Reasoning (*P* < 0.001, *df* = 12, *t* = 4.91). They also improved slightly on Reading Comprehension (*P* = 0.03, *df* = 12, *t* = 2.45). LSAT improvement was significantly correlated with the reasoning subtest scores (LG: *R* = 0.85, *P* = 0.0002; LR: *R* = 0.68, *P* = 0.01), but not with RC (*R* = 0.5, *P* = 0.08), suggesting that changes in LSAT scores were driven by reasoning gains.

### Changes in diffusion measures

The trained and control groups did not differ at time 1 on any of the diffusion measures (FA, RD, AD, and MD). The groups also did not differ on grey or white matter volume at either time 1 or time 2. Further, we did not observe a significant effect of training on grey/white matter classification within the trained group.

Whole-brain voxel-wise statistical analyses revealed significant decreases in RD and MD (but not in FA or AD) from time 1 to time 2 for the trained group compared to the control group, as described below. RD decreases were observed in white matter connecting frontal cortices (genu, anterior body of the corpus callosum, anterior corona radiata), and in descending white matter, including superior corona radiata, anterior internal capsule, and ventral brainstem (Figure 1, green). Training-related decreases in MD were generally more lateral, and closer to cortex, with the exception of decreases through anterior callosum (Figure 1, blue). MD decreases were particularly notable in white matter underlying left frontal cortex, including left RLPFC (see Figure 1, *Z* = 0), and right parietal cortex (Figure 1, *Z* = 30, *X* = 35).

When we extracted all four diffusion measures (FA, AD, RD, and MD) for the trained group from the voxels showing significant changes in RD (Figure 1, inset, left) and MD (Figure 1, inset, right), different patterns emerged. On average, voxels showing a decrease in RD also showed an increase in FA, which was likely not significant at the whole-brain level because of a slight concomitant decrease in AD. In contrast, voxels showing a significant decrease in MD showed roughly equal decreases in AD and RD, and therefore, no trend towards a change in FA.

Locations of RD and MD changes according to the JHU White Matter Label Atlas are shown in Tables 2 and 3, respectively. While 85% of the voxels showing a decrease in RD were classified by the JHU atlas, only 35% of the voxels showing a decrease in MD fell into a white matter label, likely because this atlas classifies primarily deep white matter and not white matter nearer to cortex. Importantly, because TBSS analyses test voxels along a white matter skeleton, we tested only voxels that were solidly in white matter, and not those contaminated by gray matter.

**Table 2:** **Locations of voxels showing RD decreases**.

**White matter label**	**Number of voxels**
Anterior limb of internal capsule, *R*	526
Genu of corpus callosum	332
Superior corona radiata, *R*	254
Body of corpus callosum	178
Cerebral peduncle, *R*	178
Anterior corona radiata, *R*	172
Superior corona radiata, *L*	170
Anterior corona radiata, *L*	136
Corticospinal tract, *R*	133
Posterior limb of internal capsule, *R*	75
Superior cerebellar peduncle, *R*	68
Superior fronto-occipital fasciculus, *R*	67
Middle cerebellar peduncle	54
Posterior corona radiata, *R*	41
Pontine crossing tract	37
Splenium of corpus callosum	37
Medial lemniscus, *R*	13
Superior longitudinal fasciculus, *R*	5
External capsule, *R*	2
Total labeled voxels	2478
Total voxels	2912

Voxels are 1 mm^3^. L = left, R = right.

**Table 3:** **Locations of voxels showing MD decreases**.

**White matter label**	**Number of voxels**
Anterior corona radiata, *L*	468
Superior corona radiata, *L*	299
Superior longitudinal fasciculus, *R*	266
Genu of corpus callosum	253
Body of corpus callosum	224
Superior longitudinal fasciculus, *L*	100
External capsule, *R*	99
Superior corona radiata, *R*	37
Uncinate fasciculus, *R*	13
Anterior limb of internal capsule, *L*	3
Superior fronto-occipital fasciculus, *L*	1
Total labeled voxels	1763
Total voxels	4989

Voxels are 1 mm^3^. L = left, R = right.

**Figure 2:**
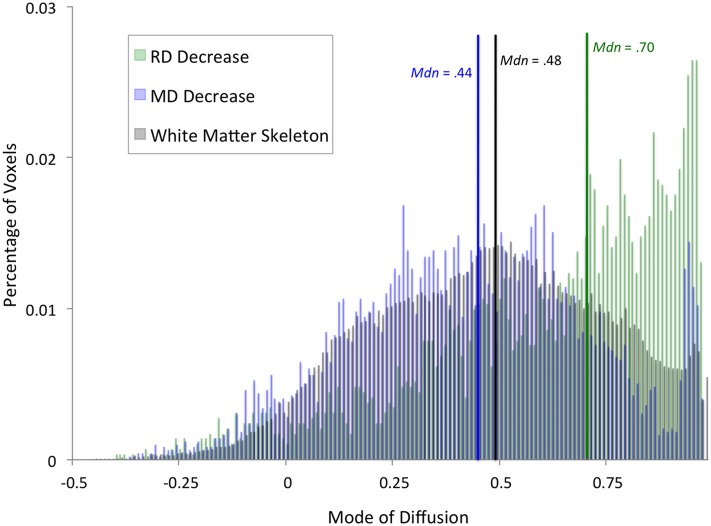
**Distribution of diffusion mode for whole-brain results.** Histograms showing percentage of voxels with a given mode value were calculated for voxels that exhibited a significant training effect at the whole-brain level in RD (green) or MD (blue). For comparison, a histogram of mode values for the entire white matter skeleton is shown (black). Median mode for each set of voxels is marked by a vertical line (RD: green, MD: blue, white matter skeleton: black).

In addition to an apparent difference in spatial distribution, we were interested to know whether the distribution of mode of anisotropy, a proxy measure for the presence of crossing fibers, differed between voxels showing a significant decrease in RD and MD. Figure 2 shows histograms of mode values for voxels showing changes in RD (green) and MD (blue). Mode values for the entire white matter skeleton are shown for comparison (black). Voxels showing a decrease in RD have a median mode (Mdn = 0.70) that is significantly greater than the median mode of the white matter skeleton (Mdn = 0.48, Mann–Whitney U = 1.19 × 10^8^, *df* = 121,805, *P* = 1.35 × 10^186^), providing evidence that RD changes occurred in highly directional tracts. In contrast, voxels showing a decrease in MD have a median mode slightly lower than the whole white matter skeleton (Mdn = 0.44, *U* = 2.78 × 10^8^, *df* = 123,882, *P* = 2.93 × 10^−21^), suggesting that MD changes were more likely to occur in regions with crossing fibers. The peak in mode values above 0.9 comes principally from voxels in the anterior callosum. Importantly, only 5% of RD results and 6% of MD results have a negative mode, so excluding these voxels from the analysis because they were not fit well by the standard linear tensor would not appreciably alter the results.

### Diffusion-behavior correlations

**Figure 3:**
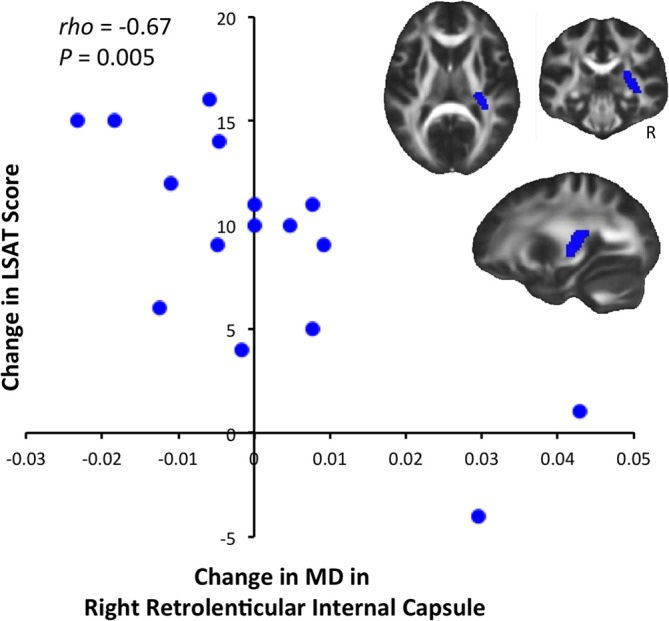
**Correlation between LSAT improvement and MD decrease.** LSAT change and MD change were significantly negatively correlated [Spearman's rho = −0.667, *P*(uncorrected) = 0.005, *P*(corrected) = 0.02] in the right retrolenticular part of the internal capsule, an anatomical ROI defined from the JHU Label Atlas. Slices shown are: *X* = 27, *Y* = −29, *Z* = 10.

We tested for significant correlations between behavioral improvement and diffusion changes at the whole-brain level, but did not find significant results (*P* < 0.05 after correcting for multiple comparisons). We then tested for correlations between diffusion and LSAT improvement within ROIs defined from the JHU White Matter Atlas. This analysis revealed a significant negative correlation between change in MD and change in LSAT score in the retrolenticular part of the right internal capsule [Figure 3, Spearman's rho = −0.667, *P*(uncorrected) = 0.005]. This correlation was significant after a randomization-based family-wise error correction for 48 comparisons (Nichols and Hayasaka, 2003), as we tested each of the regions in the JHU White Matter Label Atlas [*P*(corrected) = 0.02].

## Discussion

In this study, we sought to test whether three months of reasoning training altered white matter microstructure. While we found no changes in white matter volume, we observed training-related changes in diffusion parameters within white matter. Indeed, our results show that reasoning training led to decreased RD in white matter connecting frontal cortices, and decreased in MD in white matter underlying left frontal and right parietal cortices. These experience-dependent changes fall into tracts that would be predicted by prior work showing that reasoning relies on an interhemispheric frontoparietal network (for review, see Prado et al., 2011). Our findings are also consistent with the view that reasoning is largely left-hemisphere dominent (e.g., Krawczyk, 2012), but that homologous cortex in the right hemisphere can be recruited as needed to support complex reasoning. Perhaps learning to reason more efficiently involves recruiting compensatory neural circuitry more consistently.

Relationships between diffusion changes and LSAT changes were not particularly robust, perhaps because neuroplastic changes were driven by experience shared across individuals. We found an unpredicted negative correlation between change in MD and improvement on the LSAT in the retrolenticular part of the right internal capsule (white matter that interconnects posterior cortices and thalamus) as well as corticopontine fibers originating in the right parietal lobe (Nolte, 2009). Future research with a larger sample size will be needed to determine whether these brain-behavior correlations are replicable, and whether there are any additional statistically significant relationships between diffusion change and reasoning improvement.

The results featured here meet a more conservative criterion than several prior training studies, in that changes in the trained group needed to surpass changes in the control group to be considered significant. The participants in our study were, on average, in their early twenties, and developmental changes in white matter are known to occur during this age range (Lebel et al., 2008). Additionally, both groups consisted largely of university students, and their academic experiences over the course of 3 months alone could have altered their white matter microstructure. Thus, changes that were significantly greater in the trained group than in a well-matched control group provide strong evidence for experience-dependent plasticity, and not simply maturational changes.

An active control group is often preferable to a passive control group in training studies, because it controls for general factors like beliefs about how much one is learning or improving on a task. For this study, however, selecting an appropriate active control group for this study would have been difficult as most adults would not choose to spend 100 hours over 3 months training on a skill that is not directly relevant to their life goals. Had we administered an artificial active control training program in the lab, differences between groups in neuroanatomical changes could have been confounded by differences in levels of motivation and attention. Alternatively, if our control group had consisted of individuals enrolled in a different professional training course, such as the Medical College Admission Test (MCAT), we might have encountered initial group differences based on differences in interests, coursework, and experiences that would predispose students to seek admission to one professional program over another.

In this paper, we have examined changes in four measures of diffusion. On the one hand, this broad approach introduces a multiple comparison problem that would not exist if we had simply investigated changes in a single measure. On the other hand, if we had only looked at one measure, we would have painted a limited picture of white matter plasticity. Further, we did not have strong reason to believe that one index of white matter microstructure was more important or more likely to change with training than the others.

It is important to recognize that a tract defined by an atlas does not necessarily reflect an individual's anatomical tract. Rather, it reflects where tracts lie on average across individuals. At the current resolution, it is not possible to determine whether any given voxel contains axons connecting, for example, bilateral motor cortices or frontal and parietal cortices. Advances in diffusion imaging, such as diffusion spectral imaging (DSI), may make it possible to better classify the principal direction(s) of each voxel that shows a quantified change in diffusion. However, these sequences have yet to be used in the context of research on neuroplasticity. As the required scan time for advanced diffusion imaging pulse sequences decreases, and as scanners employ stronger gradients, it should become feasible to include more sensitive measures of white matter microstructure in studies of neuroplasticity that involve multiple structural and functional brain scans.

Even with advances in imaging methodology that make it possible to determine the direction of diffusion precisely, the study of white matter plasticity in humans will still be limited by the scale at which we can observe neuroanatomical changes. The cellular basis for training-induced changes in diffusion in humans is and will remain unclear, at least for the foreseeable future, though it is possible to speculate about potential mechanisms based on plasticity observed in animals (see Zatorre et al., 2012 for review).

Studies in animals have shown that both decreased RD and increased FA are related to increased myelination (Vorisek and Sykova, 1997; Zhang et al., 2009; Blumenfeld-Katzir et al., 2011). It is possible, then, that the experience-dependent decreases in RD (and increases in FA) that we observed were driven by myelination, especially because they tended to be in highly directional, heavily myelinated tracts. However, it is important to note that while myelin does affect diffusion (Mottershead et al., 2003; Concha et al., 2010), unmyelinated axon membranes do as well (Partridge et al., 2004), and myelin volume and axon counts are very highly correlated (Schmierer et al., 2007; Concha et al., 2010). Therefore, the extent to which axonal cell membranes also constrain diffusion is unclear.

Decreased MD, on the other hand, has been related to proliferation and/or growth of astrocytes (Blumenfeld et al., 2006; Sagi et al., 2012). A reduction in MD could additionally or alternatively reflect the myelination of axons traveling in multiple directions. It is therefore intriguing that we observed decreased MD near cortex, and also in white matter that was not highly directional and therefore could contain crossing fibers. Hopefully, future research linking changes in cell structure and function to plasticity in large-scale networks will further our understanding of how experience shapes the anatomy of the human brain.

### Conflict of interest statement

The authors declare that the research was conducted in the absence of any commercial or financial relationships that could be construed as a potential conflict of interest.
